# Insights into the microbial diversity and structure in a full-scale municipal wastewater treatment plant with particular regard to Archaea

**DOI:** 10.1371/journal.pone.0250514

**Published:** 2021-04-26

**Authors:** Grażyna Płaza, Łukasz Jałowiecki, Dominika Głowacka, Jakub Hubeny, Monika Harnisz, Ewa Korzeniewska

**Affiliations:** 1 Environmental Microbiology Unit, Institute for Ecology of Industrial Areas, Katowice, Poland; 2 Genomed SA, Warsaw, Poland; 3 Faculty of Geoengineering, Department of Engineering of Water Protection and Environmental Microbiology, University of Warmia and Mazury Olsztyn, Olsztyn, Poland; Free University of Bozen/Bolzano, ITALY

## Abstract

Due to limited description of the role and diversity of archaea in WWTPs, the aim of the study was to analyze microbial community structures and diversities with particular regard to Archaea in the samples taken from different stages of the full-scale municipal wastewater treatment plant and effluent receiving water (upstream and downstream discharge point). Our study was focused on showing how the treatment processes influenced the Eubacteria and Archaea composition. Alpha and Beta diversity were used to evaluate the microbial diversity changes in the collected samples. *Proteobacteria* was the largest fraction ranging from 28% to 67% with 56% relative abundance across all samples. Archaea were present in all stages of WWTP ranged from 1 to 8%. Among the Archaea, two groups of methanogens, acetoclastic (*Methanosarcina*, *Methanosaeta*) and hydrogenotrophic methanogens (*Methanospirillium*, *Methanoculleus*, *Methanobrevibacter*) were dominant in the technological stages. The obtained results indicate that the treated wastewater did not significantly affect eubacterial and archaeal composition in receiving water. However, differences in richness, diversity and microbial composition of Eubacteria and Archaea between the wastewater samples taken from the primary and secondary treatment were observed.

## Introduction

Despite the advantages of culture‐dependent techniques, including low cost and the potential to combine with other methods, the availability of culture‐based methods for studies of environmental microbes significantly reduces the research on microbial community structure in environmental ecosystems [1]. Culture-independent molecular methods based on 16S rRNA genes and on sequencing of total DNA (metagenomic sequencing) have been developed to characterize the phylogenetic and functional diversity of microbial communities. Metagenomic tool is useful in identifying the microbiome structure of various environments, including sludge and wastewater samples. Recently, integrated “omics” analyses have provided an enhanced understanding of the species and their functions in wastewater microbial systems [2–7]. However, there are still many gaps in our knowledge on the phylogenetic changes of the microbiome during the technological process. The deep knowledge on changes of microbial structure in biological treatment plants is needed to improve the technological stages of the treatment, and to better understand the function and role of microbiome in biological wastewater treatment technology.

The wastewater is treated through multiple aerobic and anaerobic processes of microbial metabolisms. Archaea are one of the groups of microorganisms which have a key contribution to wastewater treatment. Ammonia-oxidizing archaea (AOA) are the core component of nitrogen transformation in wastewater treatment processes. The abundance of ammonia-oxidizing bacteria (AOB) and ammonia-oxidizing archaea (AOA) and their amoA genes in the samples taken from the aerobic activated sludge tanks, recycled sludge and anaerobic digesters of a full-scale wastewater treatment plant was determined by Islam *et al*. [8]. They used polymerase chain reaction and denaturing gradient gel electrophoresis to generate diversity profiles of two groups microbes. The results obtained by the authors suggest that these two populations may have a cooperative relationship for the oxidation of ammonia. The ammonia-oxidizing bacterial was dominated in the aerobic tanks. While the AOA was abundant in the digesters. Zheng *et al*. [9] investigated the transcriptional abundance and community structure of both microbial populations, ammonia-oxidizing archaea and ammonia-oxidizing bacteria in 14 WWTSs using amoA genes as molecular markers. The coexistence of AOB and AOA has been confirmed in WWTSs, although which is more dominant remains a matter of debate. As presented in the literature AOB were the dominate microbes in most municipal and industrial wastewater treatment plants [10,11]. In contrast, dominance of AOA has been found in some WWTPs under extreme conditions, such as moderate toxicity or low temperature, as well as in nitrifying trickling filters and moving bed bioreactors [12,13]. The reason behind this finding is that differences in process parameters and water conditions lead to a competitive relationship and different niches between AOB and AOA in different WWTPs, which impact the prevalence of AOA or AOB [11,14]. Currently there is still a lack of information on the prevalence and role of AOA in ammonia oxidation in the treatment of municipal wastewater. According to the literature the presence of AOA appears to be dependent upon oxygen concentration and sludge retention time, however, the role of AOA and their function in activated sludge systems have not yet been fully elucidated. Little is presently known on their abundances and community structures, and what environmental factors influence on their survival and diversity. As noted the Archaea are also essential in converting pollutants into environmentally friendly products for wastewater treatment [15,16]. However, compared with bacteria which are widely studied in wastewater treatment systems, the characteristics and contributions of Archaea are still not well known. Neither ecological patterns of Archaea in the complex wastewater microbiome, nor the metabolisms of certain members of archaeal community, are fully understood. One of the reasons of the situation is the fact that most of the species of archaeal population are not culturable.

In this context, the purpose of this study was to analyze microbial community structures and diversities with particular regard to Archaea in the samples taken from different stages of the full-scale municipal wastewater treatment plant in the Silesian Region (Poland) by Illumnia HiSeq platform. The study facilitated the evaluation of similarities and differences in Eubacteria and Archaea composition during the wastewater treatment process and their changes in effluent receiving water. This research may further elucidate the bacterial and archaeal structures and can aid in developing promising strategies and in proper management technologies for wastewater treatment plants.

## Materials and methods

### Description of WWTP and sample collection

Wastewater, sludge and receiving water samples were collected from the full-scale municipal wastewater treatment plant in one of the largest urban areas in the EU and the center of Poland’s industries, particularly coal and metal, with a density of 1,600 people per km^2^ (geographical coordinates: N 50° 5ʹ 35.881; E 19° 3ʹ 32.202). No specific permissions were required for the locations/activities of sampling. The field studies did not involve endangered or protected species. In 2018, WWTP had a population equivalent of 189,332 inhabitants, the average flow rate was 26,830 m^3^d^-1^ and the plant was operated with a hydraulic retention time (HRT) of ~12 h and a solid retention time (SRT) of 25 days. The wastewater treatment is carried out in mechanical, biological, and chemical processes in the form of phosphorus precipitation. The detailed description of the wastewater treatment plant (WWTP) and its technological parameters are presented by [17].

**Fig 1:**
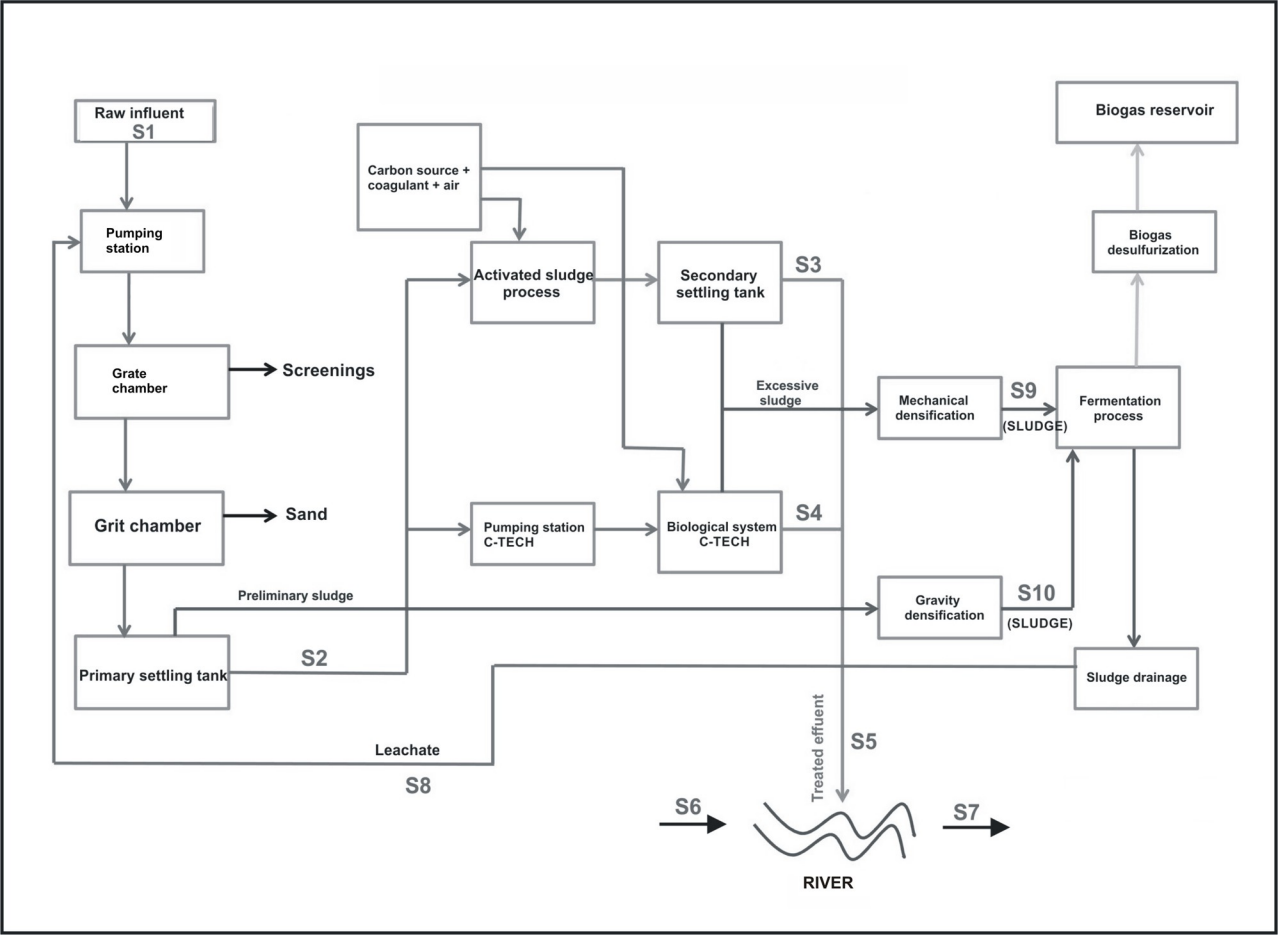
Scheme of municipal wastewater treatment plant with the samples localization. S1—Untreated wastewater; S2—Wastewater after the primary settling tank; S3—Wastewater after the secondary settling tank; S4—Wastewater after the selector and C-TECH reactor; S5—Treated wastewater; S6 –River water before the discharge of treated wastewater; S7 –River water after the discharge of treated wastewater; S8—leachates; S9—Sludge after the mechanical compression; S10—Sludge after the gravity compression.

30 grab samples were collected from the various stages of technological process of municipal wastewater treatment plant (Fig 1). Samples of river water were collected 100 m upstream and downstream from the wastewater discharge point. The samples were collected over three sampling campaigns in June and November 2018, and in March 2019. They were collected in triplicate and placed in sterile bottles in volumes 1–2 liters and then, they were transported to the laboratory on the same day. DNA extraction was done immediately after transportation.

The results of the study are presented as average values from the three sampling campaigns.

### DNA extraction, PCR amplification and Illumina sequencing

The wastewater samples in volumes between 10 mL and 400 mL depending on the sampling point were filtered in triplicate through 0.22 μm micropore membrane (Whatman, UK) and kept in −80°C until DNA extraction. 0.25 g of sludge samples were used for DNA isolation. Genomic DNA was extracted by the commercial kits following the manufacturer’s instruction. The Power Water kit (MoBio Laboratories Inc., CA, USA) and the Power Soil kit (MoBio Laboratories Inc., CA, USA) were used for wastewater/water and sewage sludge samples, respectively. Finally, a DNA pool from the triplicates was prepared resulting in one DNA extract per sample. The quality of DNA degradation was determined by running in 1% agarose gel. The concentration and purity of DNA (A260/280 and A260/230 ratios) were determined by microspectrophotometry (BioSpectrometer, Eppendorf).

Extracted DNA samples were sent to Macrogen Inc. (Seoul, South Korea) for library preparation and sequencing. Samples were quantified by the picogreen method using Victor 3 fluorometry and once again their quality was assessed by gel electrophoresis (1% agarose gel, 30 min running at 160V, 1ul of DNA loaded). Illumina TruSeq DNA PCR-Free libraries were prepared manually following the manufacturer’s protocol TruSeq DNA PCR-Free Sample Preparation Guide, Part #15036187 Rev. D (Illumina, San Diego, CA, USA). Libraries were normalized to 4 nM, pooled at equal volumes, and sequenced using Illumina HiSeq sequencing system. The DNA was mechanically sheared by sonication to reach the average insert size of 350bp. Then, the ends were repaired, 3`adenylated, and the adapters were ligated. The ready libraries were tested using LightCycle qPCR and the size distribution was assessed by Agilent Technologies 2100 Bioanalyzer using a DNA 1000 chip and sequenced on NovaSeq6000 in an S4 flowcell lane using 2x150bp configuration. The library quantity was assessed using qPCR assay following the Illumina qPCR Quantification Protocol Guide (Part # 11322363 Rev. C). The following primers were used: qPCR primer 1.1: 5ʹ AATGATACGGCGACCACCGAGAT 3ʹ qPCR primer 2.1: 5ʹ CAAGCAGAAGACGGCATACGA 3ʹ and the both primers were HPLC purified. The following thermal profile was used: hot start 95°C 3 minutes, 10 cycles of 95°C 3 seconds, 60°C 30 seconds.

### Data analysis

Sequencing results were uploaded to the MetaGenome Rapid Annotation Subsystems Technology (MG-RAST version 4.0.3) server as FASTQ files for analysis [18]. Default parameters were used for all software unless otherwise specified. Pre-process of raw reads involved removing adapter sequences using skewer program Each file underwent quality control (QC) including quality filtering (removing sequences with ≥5 ambiguous base pairs) and length filtering (removing sequences with a length ≥2 standard deviations from the mean) using Fastq-Mcf program v.0.11.9.). Also, the dereplication process was performed. The identification of the protein coding sequences was then carried out using the FragGeneScan program (9) filtering out putative protein sequences overlapping ribosomal RNA sequences and clustering the sequences with 90% similarity (cd-hit). The representative sequences of individual clusters were used to assign taxonomies based on the RefSeq database.

Alpha diversity (α) was used to analyze the diversity of a population within both Eubacteria and Archaea communities. Beta diversity (β) was used to evaluate the differences in microbial composition between the samples.

The metagenomic analysis were performed in R program. The alpha diversity analysis was performed using the phyloseq package. Beta diversty analysis was performed on the basis of the Bray-Curtis measure and with the vegan package, while the clustering of the samples was performed using the UPGMA method. The graphs were generated using the ggplot2, gplots and ggbiplot packages.

The Illumina metagenomic raw sequences were submitted to NCBI database with BioProject ID: PRJNA666519 (title: Metagenomics profiling of antibiotic resistance genes and mobile genetic elements).

## Results and discussion

### Eubacterial and archaeal structure analysis

**Fig 2:**
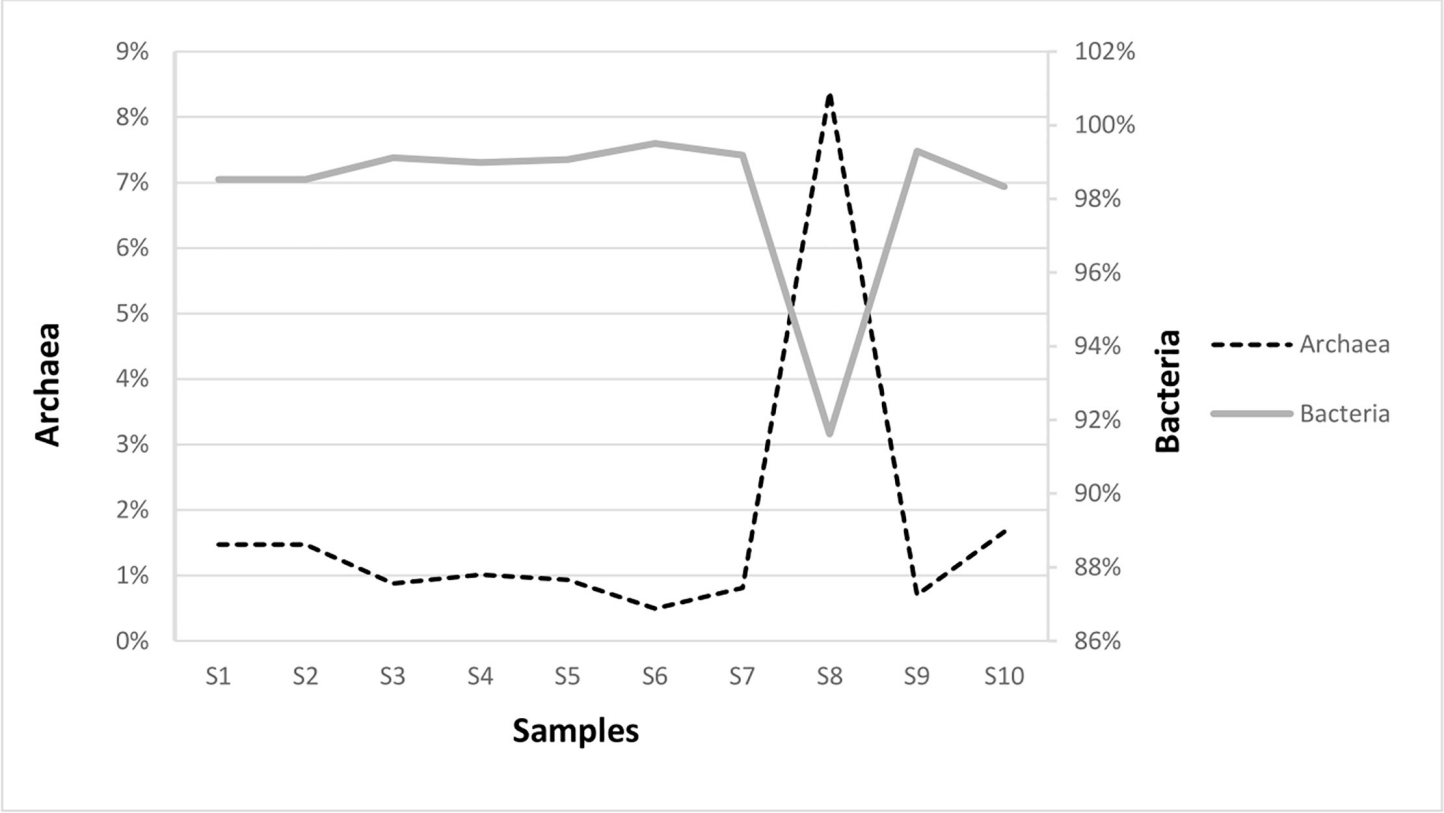
The percentage of Eubacteria and Archaea in the collected samples.

The metagenomes of Eubacteria consisted of 476 million paired-end reads and ranged from 13 million to 86 million reads across the ten samples, with the most reads in samples: S1 (76 million), S6 (54 million), S9 (56 million), and S10 (86 million). The average was 48 million reads per sample. For archaeal sequences a total of 7 million paired-end reads across the ten samples were obtained. Most of the reads were noted in S4 and S8 samples: 650,450 and 510,776 respectively. In the leachate from anaerobic digester (S8 sample, see Fig 1), the relative abundance of Archaea was the highest and researched 8.4% compared with the rest of the samples (Fig 2). Interesting, in S8 sample the values of richness and diversity indices for Archaea were lower compared to the rest of samples (Table 1). Probably the operational conditions of fermentation process like pH, salinity, temperature and lack of oxygen affected the distribution of archaeal community in the sample S8.

**Table 1:** Estimated OTU richness and diversity indices of the samples from the various stages of technological process and receiving water (above and below effluent discharge point).

Samples	Chao1		Observed		Shannon index (H’)		Simpson index (SI)		OTU	
	Eubacteria	Archaea	Eubacteria	Archaea	Eubacteria	Archaea	Eubacteria	Archaea	Eubacteria	Archaea
S1	1638.478	87	1634.667	87	5.869	3.405	0.984	0.927	1634.667	87
S2	1643.861	87.333	1628.667	87.333	5.944	3.455	0.991	0.935	1628.667	87.334
S3	1549.833	84.5	1540	84.5	6.276	3.856	0.995	0.967	1540	84.500
S4	1608.917	87.667	1602.333	87.667	6.403	4.013	0.996	0.974	1602.334	87.667
S5	1528.752	80.697	1508	78.333	6.249	3.695	0.995	0.95	1508	78.334
S6	1620.704	87.333	1614.667	87.333	6.134	4.054	0.994	0.976	1614.667	87.334
S7	1636.922	88.667	1617.333	88.667	6.401	4.037	0.996	0.975	1617.334	88.667
S8	1565.62	86	1555	86	6.245	2.790	0.994	0.838	1555	86
S9	1605.965	87.333	1601.667	87.333	5.741	4.023	0.987	0.975	1601.667	87.334
S10	1632.972	87.667	1629.667	87.667	6.251	3.655	0.996	0.949	1629.667	87.667

Shannon index–higher number represents higher diversity; Simpson index–higher number represents lower diversity; Chao1 index–higher number represents higher species richness.

### Richness and diversity of eubacterial and archaeal communities

According to the OTU numbers, the eubacterial and archaeal diversity indices were calculated, encompassing community richness—Chao1 and community diversity—Shannon and Simpson indices.

The values of indexes of all the collected samples for eubacterial and archaeal communities are presented in Table 1. While, graphical presentation of the indices for eubacterial and archaeal communities is presented in S1 Fig. As is shown in Table 1, the average values of investigated Chao1, Shannon and Simpson indices are much higher for Eubacteria in all samples. Chao1 index represents the community richness, while microbial diversity was evaluated by Shannon and Simpson indices. If the indices are higher, the diversity is richer. In samples S3, S5 and S8 the average values of Chao1 index for Eubacteria exhibited lower values compared to the rest of the samples, suggesting that the samples have the lowest diversity richness for the investigated domain. The situation was different for indices for evaluating the community diversity. By analyzing the results obtained for both indices, Shannon index was a more accurate indicator reflecting the diversity differences in tested samples. In comparison, lower values of the Shannon index were achieved in samples S1, S2 and S9 for Eubacteria. For the Archaea, both Shannon and Simpson indices had significantly lower values only in one sample S8, *e*.*g*. leachate 2.8 and 0.838, respectively.

Comparing the values of Chao1 index between influent and effluent, the community richness of Eubacteria and Archaea fell under the operating conditions. However, the values of Shannon and Simpson indices were higher in the effluent for both Eubacteria and Archaea communities. The effect of treated wastewater on richness of surface water (receiving water) was observed. The community richness of Eubacteria and Archaea in receiving water downstream the discharge point of treated wastewater was higher than in the surface water upstream the discharge point of treated wastewater. In the case of diversity, the treated wastewater also influenced diversity in the receiving water. The higher values of Shannon and Simpson indices were noted upstream the discharge point of treated wastewater. However, in the case of diversity indices for Archaea their values were similar for samples taken both upstream and downstream the effluent discharge point. According to the indices values the investigated samples had high microbial diversity compared with the literature data [19,20].

The difference in microbial community diversity associated with stages of wastewater treatment, was estimated based on phylogenetic distance metrics using Bray-Curtis beta diversity. The differences in the composition of Eubacteria communities in different stages visualized by heatmap and PCA are presented in S2 Fig. Beta diversity analysis revealed that the samples were clustered into one main group consisting of the samples collected during and after treatment (S3, S4, S5), and upstream (S6) and downstream (S7) the effluent discharge point to the river. The cluster community had the maximum variation in 71.3% (PC1) and 15.7% (PC2). The samples clustered within the group were positioned at close distance to each other, showing a similar eubacteria community composition. Whereas the rest of the samples, *e*.*g*. S1, S2, S8, S9 and S10 were clearly different from them, and indicated specific microbial community. Taking into account the obtained results, the treated wastewater did not significantly affect eubacterial composition in the receiving water.

**Fig 3:**
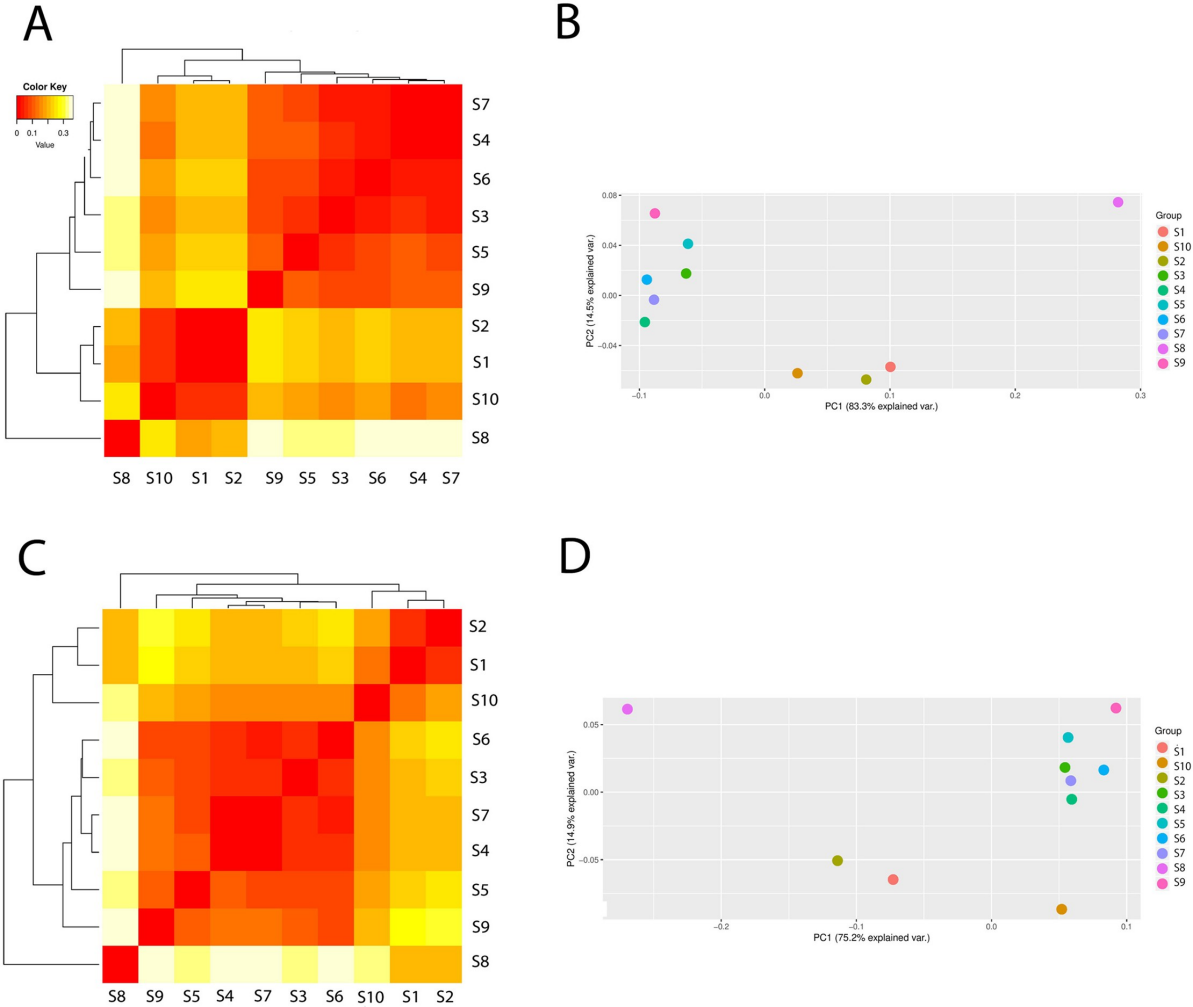
The differences in archaeal composition (beta diversity head map) and principal component analysis (PCA) based on the operational taxonomic unit abundance calculated by Bray-Curtis distance matrices, and presented at class level (A and B) and order level (C and D).

The differences in Archaea composition between the samples with the PCA are presented in Fig 3. In the case of Archaea, the samples could be clustered into two main groups: one group contained the following samples: S3, S4, S5, S6, S7, S9 and the second one–S1, S2, S10. The sample S8 (leachate) was very distant from the two groups which suggested that the leachate from the fermentation process and biogas production were completely different in archaeal community composition. Similar results was obtained from analysis of richness and diversity indices (Table 1). As can be seen from the PCA analysis the samples clustered together were positioned at a very close distance to each other (Fig 3). As presented, the treated wastewater did not influenced on the archaeal composition in surface water.

**Fig 4:**
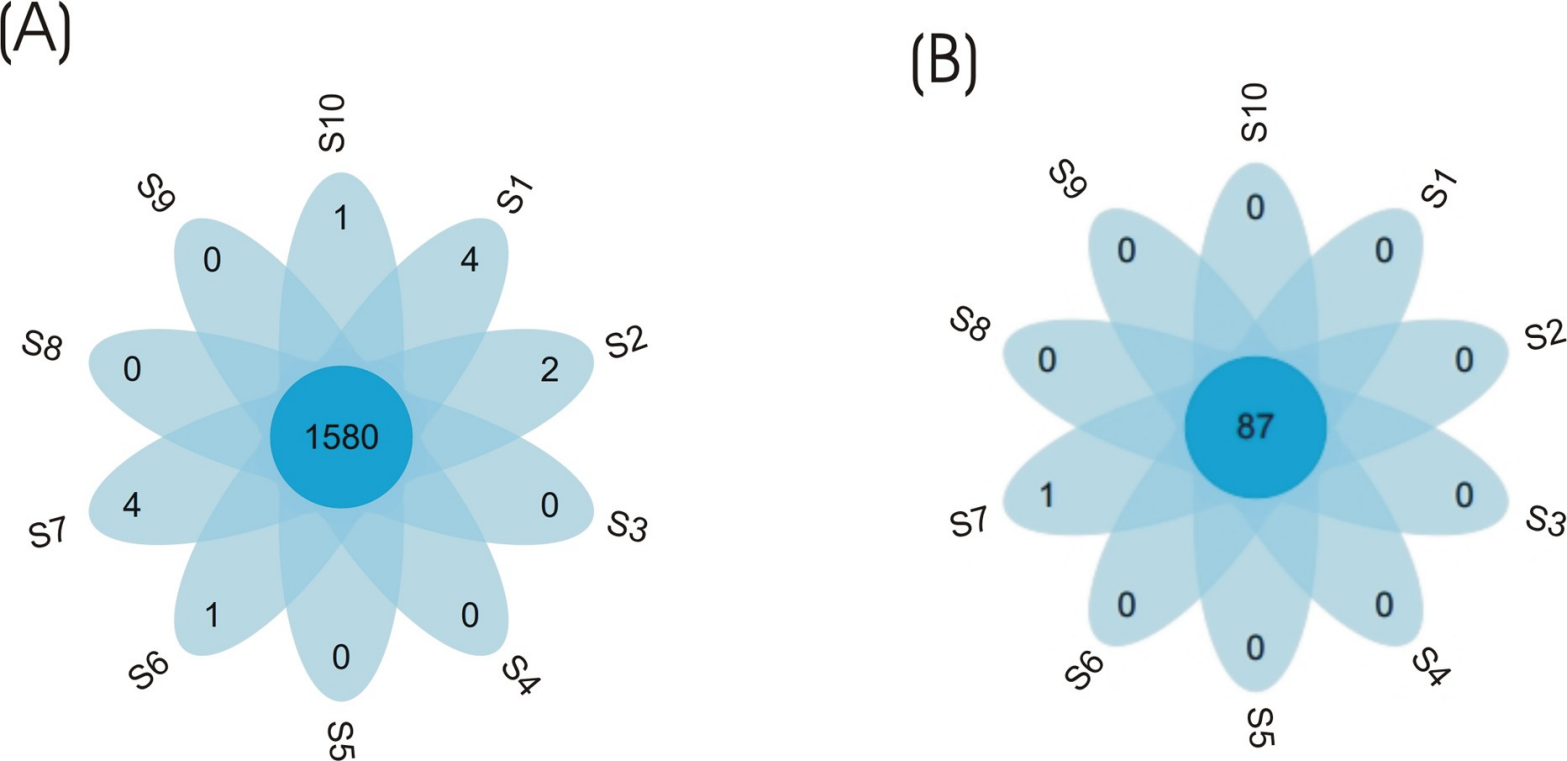
Petal flower map-based operation taxonomic units for eubacterial and archaeal communities. Each petal in the petal map represents one sample. The middle core number represents the common OTUs of all samples and the number on the petals represents the unique OUT number of each sample.

Additionally, the petal flower diagram was used to visualize dissimilarity among eubacterial and archaeal communities in the tested samples, and indicated that the highest number of OTU similar sequences was detected in both eubacterial and archaeal communities (Fig 4).

The different values of indices detected could be caused by the environmental variables such as temperature, dissolved oxygen, conductivity, pH, HRT, SRT, influent BOD and influent total nitrogen [19–21]. While, the treated wastewater affected the eubacterial and archaeal compositions in the receiving water in lesser extent.

### Phylogenetic analysis of eubacterial and archaeal communities

Phylogenetic analysis of the eubacterial communities showed that *Proteobacteria* was the largest fraction ranging from 28% to 67% with 56% average abundance across all samples, followed by *Bacteroidetes* (ranging from 8.5% to 22%), *Firmicutes* (ranging from 3.87% to 24%) and *Actinobacteria* (ranging from 1.8% to 9.7%). Actinobacteria dominated in the surface water (samples S6 and S7) across all the samples. As reported in the literature, the phylum *Proteobacteria* dominated in municipal WWTPs followed by other groups such as *Bacteroidetes*, *Acinobacteria*, *Firmicutes* but the proportion between the groups depends on many factors such as type of sewage treatment plants, technology used, composition of influent, hydraulic configuration, *etc*. [2,19,21–23]. As presented by Yang *et al*. [20] *Proteobacteria* phylum was dominant not only in activated sludge from municipal WWTP but also in industrial WWTPs [4]. The *Proteobacteria* members encompass enormous morphological, physiological and metabolic diversity, and they are involved in carbon, nitrogen and sulphur cycles. Among the *Proteobacteria*, β-*Proteobacteria* was the most abundant class ranging from 11 to 40% with average 24.2%. The Gammaproteobacteria was the second dominant class, accounting from 6.12 to 25.5% followed by Alphaproteobacteria comprising 4.07–15.20%. Within the Firmicutes, the most abundant class was *Clostridia* ranging from 2.3% to 14.6% (average value– 8%). The result was consistent with other studies concluding that Betaproteobacteria was the dominant class in the different stages of technological process of WWTPs [24–26]. Among the orders of betaproteobacteria, *Burkholderiales* was the dominant order between 7.02 and 21.6% with average 14.6%. Our results showed that 234 families were detected across the samples, but 18 families were the most abundant across the samples. 627 genera were detected in the all samples, including 60 dominant ones. The differences in phylogenetic structure of eubacterial community between the WWTP stages are presented in S3 and S4 Figs.

So far, a few metagenomic studies were performed on full-scale WWTPs and most of them focused on bacterial community [27,28]. Recent advances in molecular-based methodologies have significantly increased our knowledge on the eubacterial phylogenetic and functional diversity of wastewater treatment systems [21], while archaea have been largely neglected. Archaea constitute a minor but constant and integral part of wastewater treatment technologies and are mainly involved in anaerobic digestion processes.

Archaeal sequences were assigned to 5 phylum, 9 classes, 16 orders, 25 families, and 59 genera. The unclassified sequences in the total community were from 5.8% in the class level to 2.7% in the genus level.

**Fig 5:**
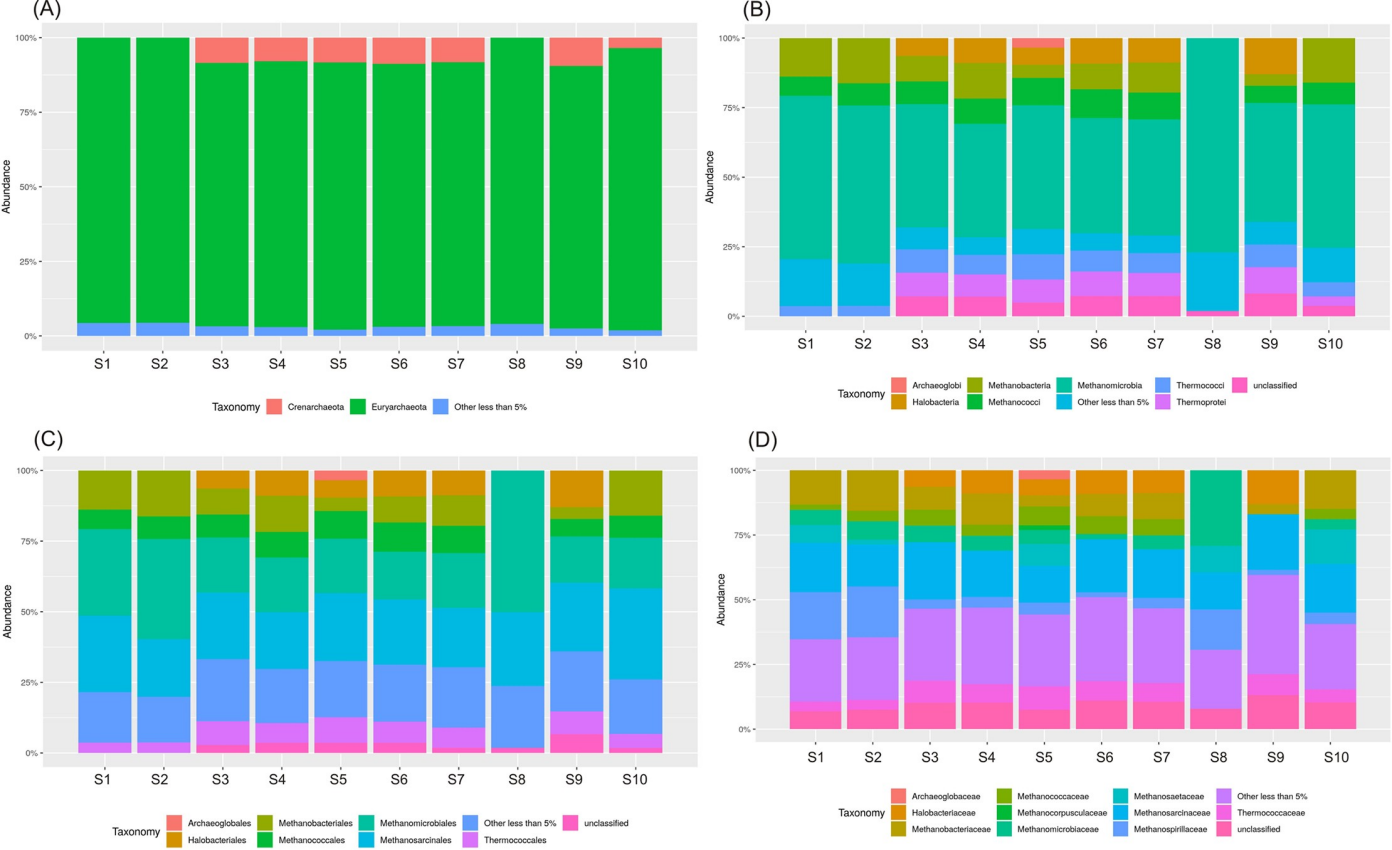
Changes of archaeal community composition at the (A) phylum, (B) class, (C) order, (D) family levels in the different stages of technological process.

*Euryarchaeota* phylum constituted the largest part of the Archaea in all the samples, and ranged from around 88% in most samples (S3-S7, S9) to 96% in leachate (S8) with the average 91.4% (Fig 5). *Crenarchaeota* was the second most abundant phylum ranging from 3.5% in the raw wastewater (sample S1) to 9.5% in the sewage sludge from the outlet of the mechanical concentrator (sample S9) with average 6.6%. *Euryarchaeota* was also detected by Illumina MiSeq sequencing approach as the predominant phyla in tannery WWTP [29] and in other previous studies on activated sludge WWTPs [30,31]. Ma *et al*. [29] compared the diversity and richness of the tannery wastewater with the municipal wastewater, and concluded that the two types of wastewater were similar in composition and structure.

**Fig 6:**
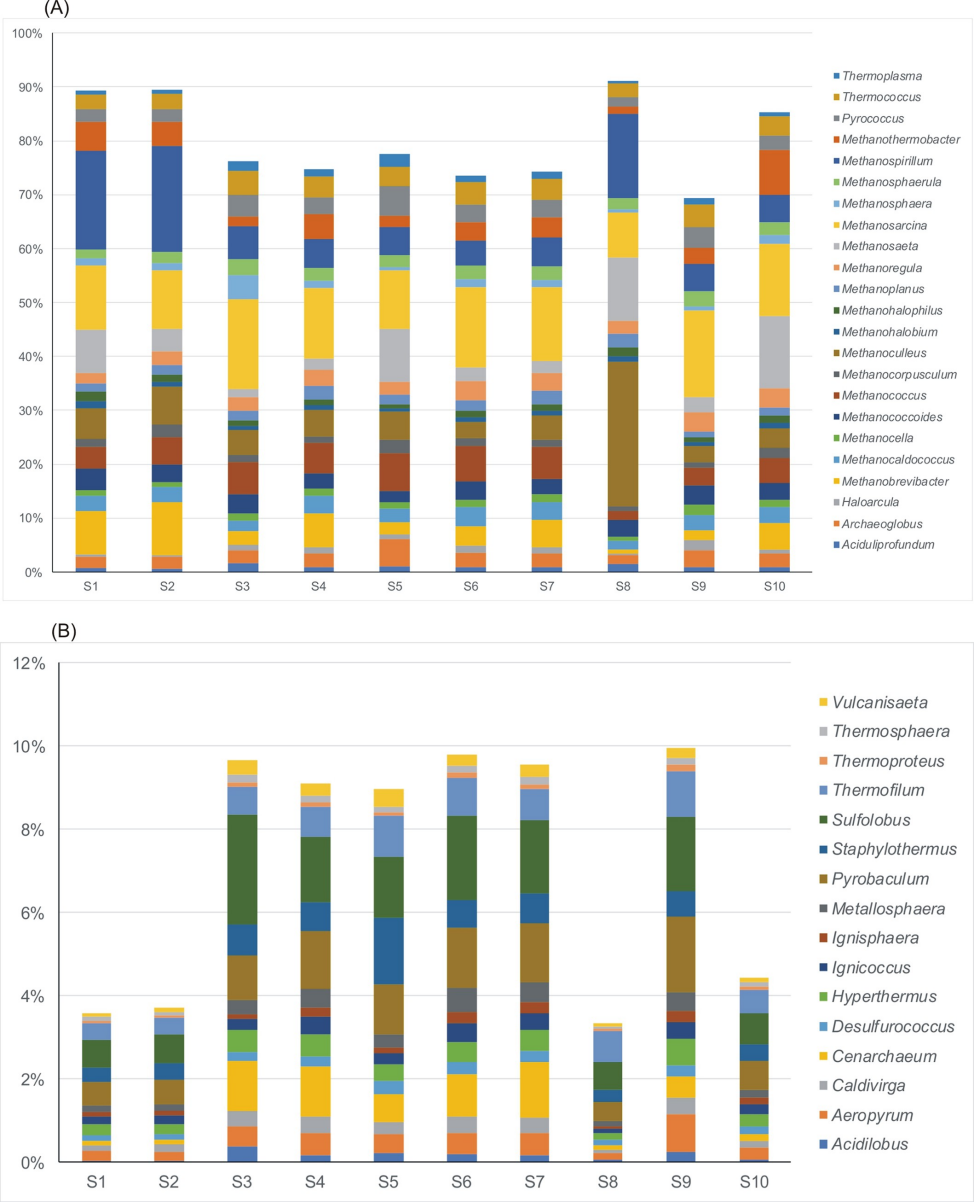
Relative abundance of dominant genera of archaeal community in various samples taken from wastewater technological process. A–Euryarchaeota, B–Crenarchaeota. Only the Euryarchaeota genera constituting more than 1% average value of the detected community in all samples are presented.

The *Methanomicrobia* was the most abundant class (41–77%) among the *Euryarchaeota* with the average value of 50% across all the samples (Fig 6A). It was dominant in the leachate (sample S8). Also, the *Methanococci*, *Methanobacteria*, and *Halobacteria* classes were detected in relatively large quantities. Within *Euryarchaeota* sixteen archaeal orders were established (Fig 6A). *Methanomicrobiales* and *Methanosarcinales* were the most dominant with very similar average values for all samples, around 24%, followed by *Methanobacteriales* (10.5%) and *Methanococcales* (7.95%). Archaeoglobales, Thermococcales, and Halobacteriales were detected in small quantity (less than 1%). Among 15 families identified Methanosarcinaceae (18.3%), Methanobacteriaceae (9.8%), Methanospirillaceae (9.01%), Methanomicrobiaceae (8.7%), and Thermococcaceae (6.9%) were dominant.

The composition of methanogens at the genus level was further investigated. Among 59 genera of archaeal community identified, only three (*Methanosarcina*, *Methanospirillium* and *Methanoculleus)* were reported as the dominant methanogens. The relative abundance of *Methanosarcinia* ranged between 8.35% in sample S8 (leachate) to 16.2% in sample S9 (sewage sludge from the outlet of the mechanical concentrator) with average 13%. *Methanospirillium* ranged from 1.81% in surface water upstream the effluent discharge point (S6) to 20% in wastewater after mechanical treatment (S2). While *Methanoculleus* was the dominant genus in leachate S8 (26.7%) with average value of 5.2% across all the samples. Also, *Methanosaeta*, *Methanococcus* and *Methanobrevibacter* were detected with lower average values 4.1%, 3.4% and 2.9%, respectively. *Methanosarcina* and *Methanosaeta* are well-known for utilizing acetate for methanogenesis while *Methanospirillium*, *Methanoculleus* and *Methanobrevibacter* are hydrogenotrophic methanogens [32]. Both, acetoclastic and hydrogenotrophic pathways are popular among the methanogens. *Archaeoglobus* and *Pyroccoccus* were identified only in treated wastewater (S5). This result described above was consistent with the findings from literature [32,33].

*Crenarchaeota* was the second dominant phylum in the archaeal community. 27% of genera belonging to *Crenarchaeota* were identified. *Pyrobaculum* and *Sulfolobus* were the dominant genera with mean value occurrence 1.06% and 1.4%, respectively (Fig 6B).

The changes in occurrence of dominant genera of *Euryarchaeota* and *Crenarchaeota* composed a core archaeal population under technological stages, and are presented in Figs 5 and 6. The composition of the original wastewater and technological process influenced the composition and load of archaeal community.

Methanogens were the most abundant archaeal community in the investigated wastewater treatment plant, and they were generally represented by *Methanosarcinales*, *Methanobacteriales* and *Methanomicrobiales* species. *Methanobacterium*, and *Methanosarcina* were detected as prevalent archaeal genera in diverse types of municipal wastewaters such as breweries or dairies WWTPs [15,34].

Methanogens form a highly specialized physiological group, unable to catabolize carbohydrates, proteins or organic compounds other than methanol, some secondary alcohols or formates. Aerated conditions do not fully exclude methanogens. Furthermore, several methanogens are now known not to be as sensitive to oxygen as originally estimated [15]. Indeed, several reports show that they can maintain viability and activity even in the presence of high levels of oxygen. *Archaeoglobus* is a sulphate-reducing archaea. *Pyrococcus* has similar characteristics to *Archaeoglobus*, and *Methanococcus* in the respect that they are all thermophilic and anaerobic. *Pyrococcus* differs, its optimal growth temperature is nearly 100°C. Unlike other archaea, *Methanoculleus* and some species of related genera can use ethanol and some secondary alcohols as electron donors as they produce methane.

In the review of Ferrera and Sanchez [21] some results of molecular studies on Archaea detection in various types of wastewater treatment system are presented. As described in literature, Archaea have been detected by the 454 pyrosequencing and Illumina, in the following samples from WWTPs: activated sludges, anaerobic sludges, effluent from swine WWTPs, anaerobic digester sludge, sludge from anaerobic reactors [10,11,29,34–39]. Our results are the first to describe the changes of archaeal communities under the technological process in an operating full-scale municipal wastewater treatment plant by the Illumnia HiSeq platform (metagenomic shotgun sequencing).

Despite their much smaller quantity, Archaea are responsible for important functions in nitrogen and carbon cycles, and methane production and finally affect the efficiency and quality of the wastewater treatment processes [40]. Some studies suggest that they might have other functions such as contributing to floc structure or being in symbiotic relationship with bacteria [41,42]. They can increase biological activities like nitrification and denitrification in aerated bioreactors of WWTPs. Some authors suggest that methanogenic archaea constantly occur in anoxic microenvironments of aerobic activated sludge [30,31]. In addition, the methane production promotes the growth of methanotrophic bacteria [31]. The degradation of some wastes, such as phtalate isomer-containing wastewaters, is accomplished by syntrophic cooperation between several different kinds of bacteria, for instance bacteria that produce hydrogen or formate coupled with *Methanospirillum* sp., which next consumes hydrogen and formate [43,44]. Treating waste anaerobically can sometimes be more cost efficient than treating waste aerobically because the aeration process uses a lot of energy and is no cost-effective.

Recent molecular studies have highlighted the presence of ammonia-oxidizing archaea (AOA) and *amo*A-encoding archaea (AEA) which are involved in wastewater treatment technologies [10,15,45–49], however the knowledge on distribution of archaea in full-scale WWTPs is still poor. Due to potentially important roles of Archaea, it is necessary to characterize the archaeal community in WWTPs.

Moreover, it was recently reported that competition and partitioning between ecological niches among phylogenetically different populations of Eubacteria and Archaea were caused by different physiological properties such as affinities for substrates, formate utilization and relationships with ammonia-oxidizing bacteria (AOB). Pan *et al*. [50] developed a mathematical model describing the microbial interaction among AOA, AOB and Anammox bacteria. The developed model could also predict and distinguish the different contributions of AOA and AOB to overall aerobic ammonia oxidizing potential. According to the model more than 50% of ammonia oxidation was mediated by AOB at initial stages. While AOA were responsible for up to 90% of the ammonium removal afterwards. Although AOB and AOA co-exist in the WWTP stages, and their relative distribution may be affected by the environmental parameters among them ammonium limitations were identified as key factor for the out competition of AOA against AOB.

## Conclusions

Recent advances in molecular-based methodologies have significantly increased our knowledge on the eubacterial phylogenetic and functional diversity of wastewater treatment systems, while archaea have been largely neglected. The results presented in this paper are the first to describe the diversity of microbial community structures with special attention to Archaea in various stages of technological process of full-scale municipal WWTP. Compared with bacteria which are widely studied in wastewater treatment systems, the distribution, structure and characteristics of archaea are still not well known and not fully understood. In our study overall view of bacterial and archaeal populations is presented. The results present differences in richness, diversity and microbial composition of Eubacteria and Archaea between the samples collected from the various technological stages. These results reveal a previously unknown diversity of Archaea in WWTP that can potentially be exploited for the development of more efficient wastewater treatment technologies. Archaea were found in low abundance from 1 to 8%) at all stages of the WWTP compared to *Proteobacteria* ranging from 28% to 67%. Phylotypes belonging to Euryarchaeota, including methanogens, were most abundant in all samples. Probably environmental and operational conditions affected distribution of microbial community structure including Archaea. The relative abundance of different types of Eubacteria and Archaea is regulated by the availability of substrates and other parameters, like temperature, pH, and salinity.

With develop of more efficient modern methods/tools more exciting break-throughs in Archaea studies in wastewater treatment from the theory improvement to technology innovation will be expected. Additional studies are necessary to fully understand microbial community diversity, distribution and functionality that can be used to develop more effective strategies for the management of wastewater treatment plant and the minimization of adverse environmental impacts. Further studies should focus on the evaluation of unclassified genera from eubacterial and archaeal communities presented in wastewater treatment plants.

## Supporting information

S1 Fig Graphical presentation of the indices for eubacterial (A) and archaeal (B) communities.
(TIF)

S2 Fig The differences in the composition of Eubacteria community (beta diversity head map) (A) with the principal component analysis; (B) based on the operational taxonomic unit abundance calculated by Bray-Curtis distance matrices, and presented at class level.
(TIF)

S3 Fig Relative eubacterial community abundance at phyla (A), class (B), and order (C) levels in the samples from different stages of technological process.
(TIF)

S4 Fig Relative abundance of dominant families constituting more than 1% of the average value (A) and genera constituting more than 0.5% of average value (B) in eubacterial community from different stages of technological process.
(TIF)

S1 File (XLSX)

S2 File (XLSX)

S3 File (XLS)

S4 File (XLSX)
