# Supplementary figures and images for: Insights into the microbial diversity and structure in a full-scale municipal wastewater treatment plant with particular regard to Archaea
Source: PLoS One. 2021 Apr 26;16(4):e0250514. doi: 10.1371/journal.pone.0250514 (PMC8075261; doi:10.1371/journal.pone.0250514)

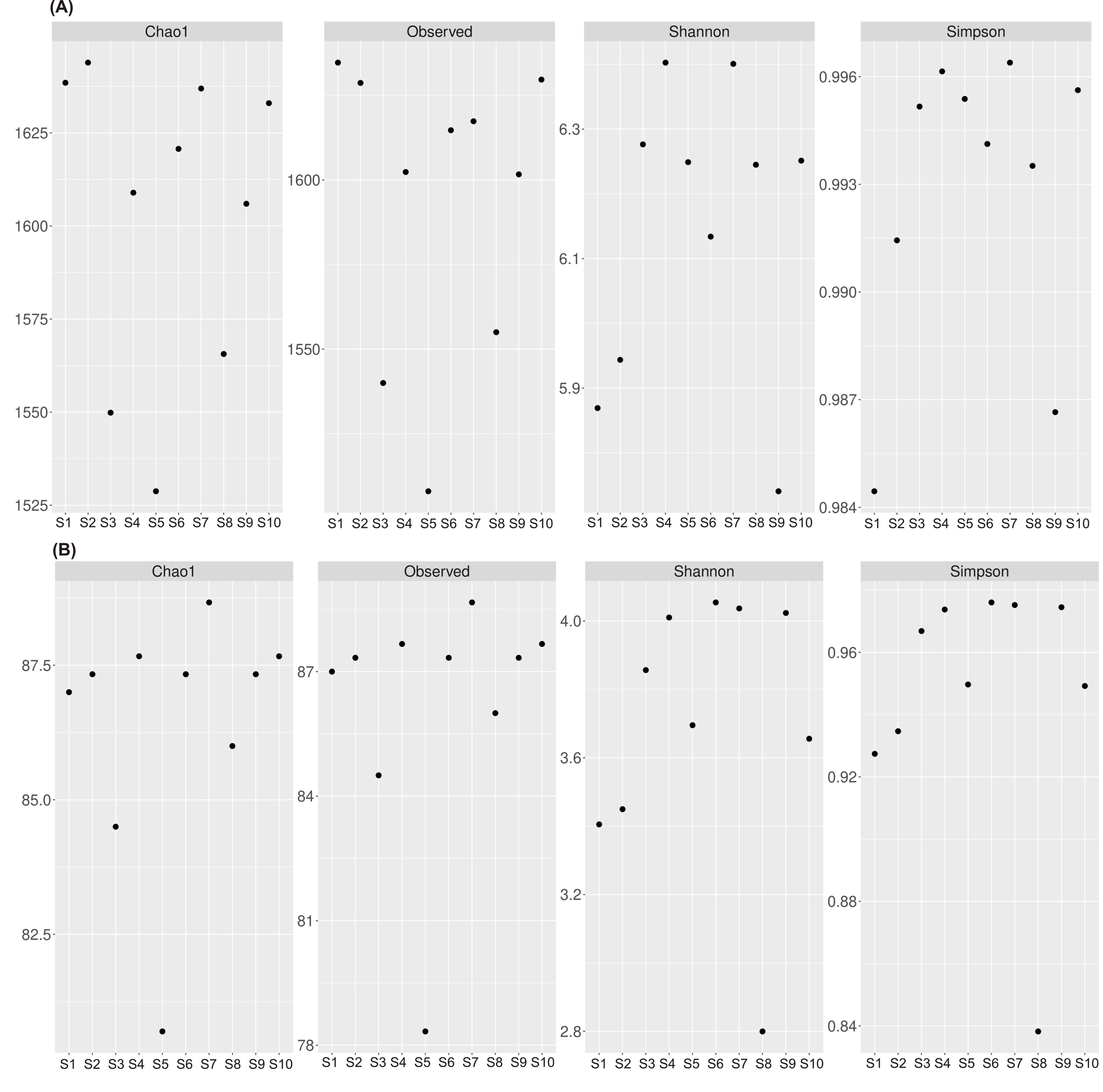

Supplement: S1 Fig — Graphical presentation of the indices for eubacterial (A) and archaeal (B) communities. (TIF) [file pone.0250514.s001.tif]

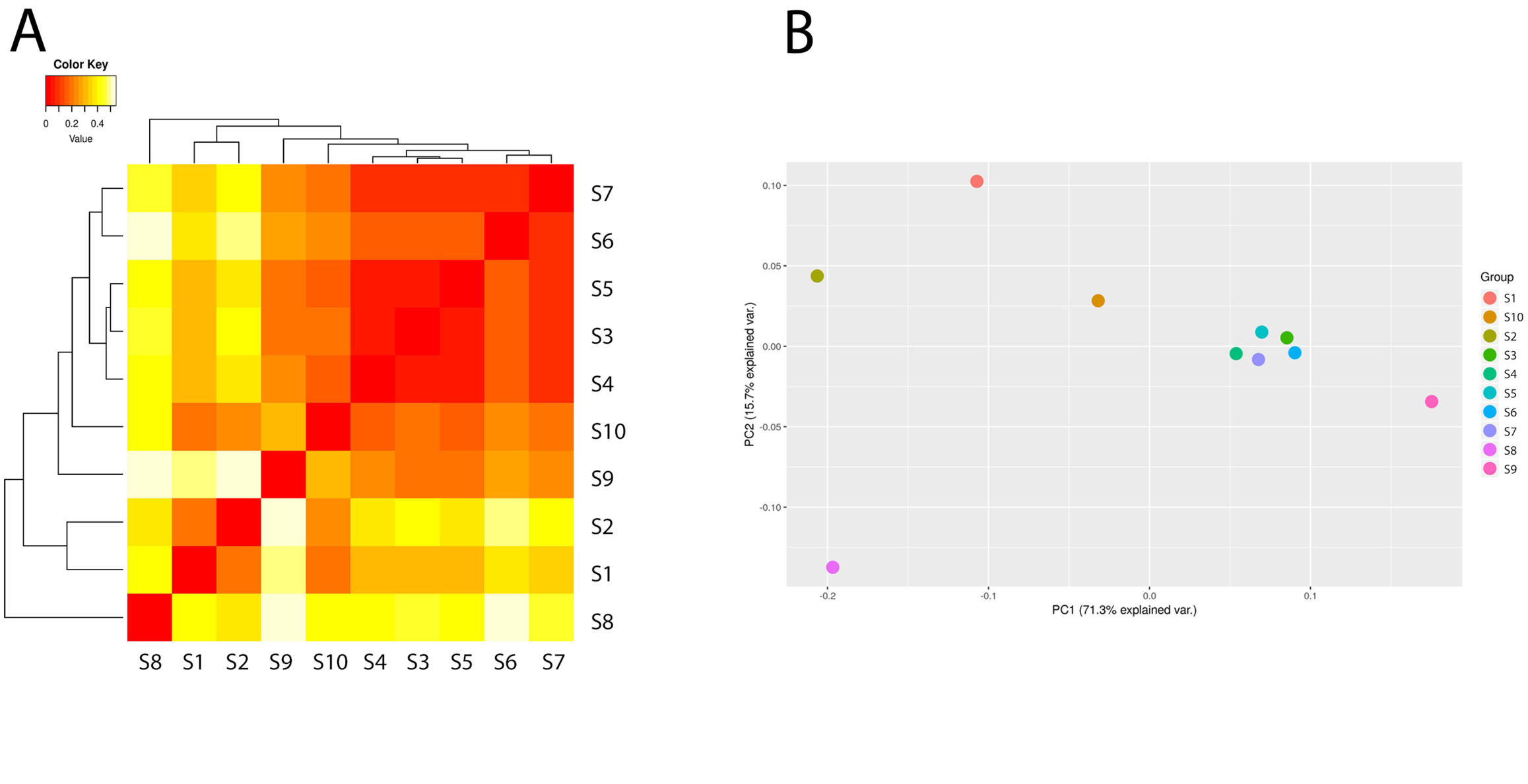

Supplement: S2 Fig — The differences in the composition of Eubacteria community (beta diversity head map) (A) with the principal component analysis; (B) based on the operational taxonomic unit abundance calculated by Bray-Curtis distance matrices, and presented at class level. (TIF) [file pone.0250514.s002.tif]

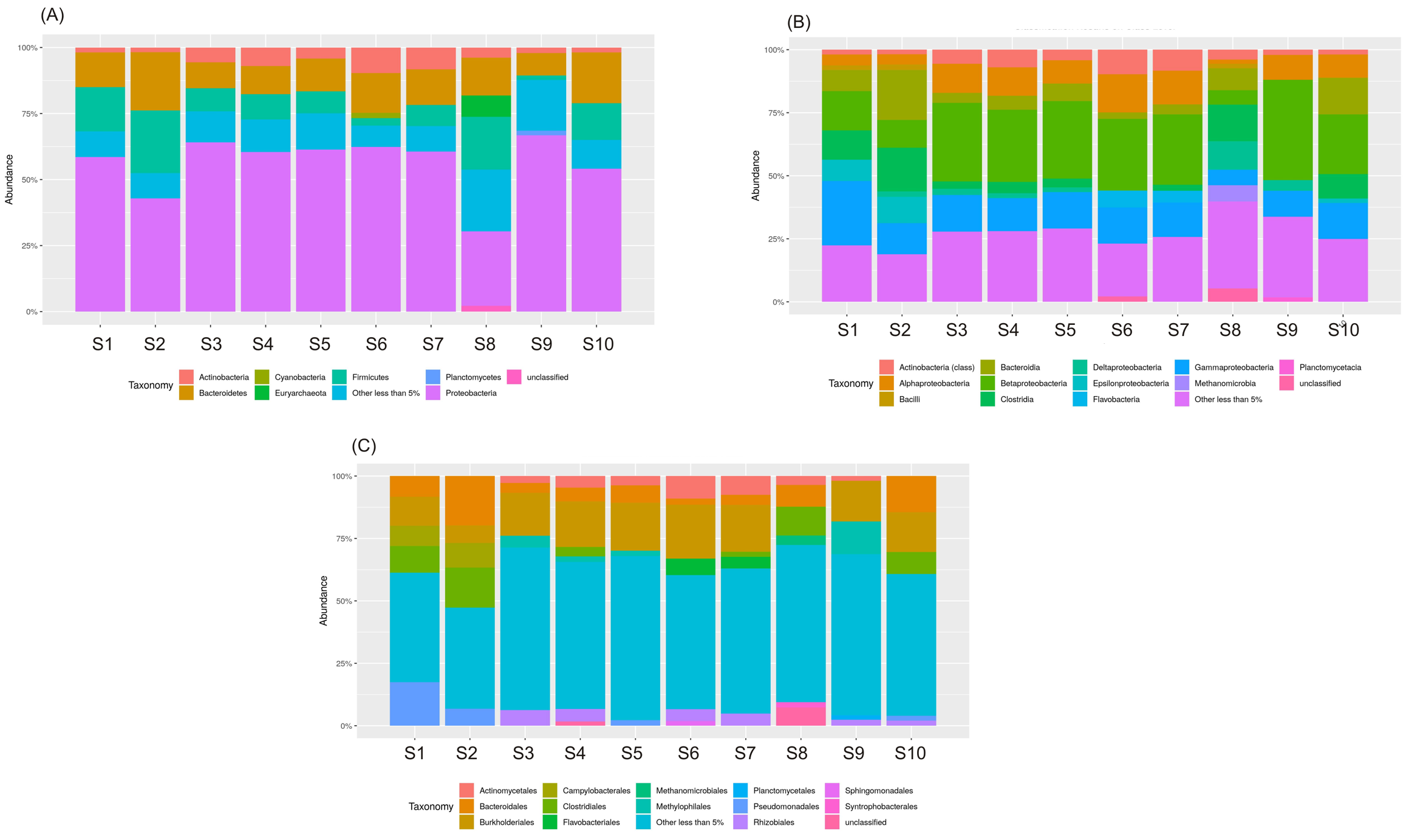

Supplement: S3 Fig — Relative eubacterial community abundance at phyla (A), class (B), and order (C) levels in the samples from different stages of technological process. (TIF) [file pone.0250514.s003.tif]

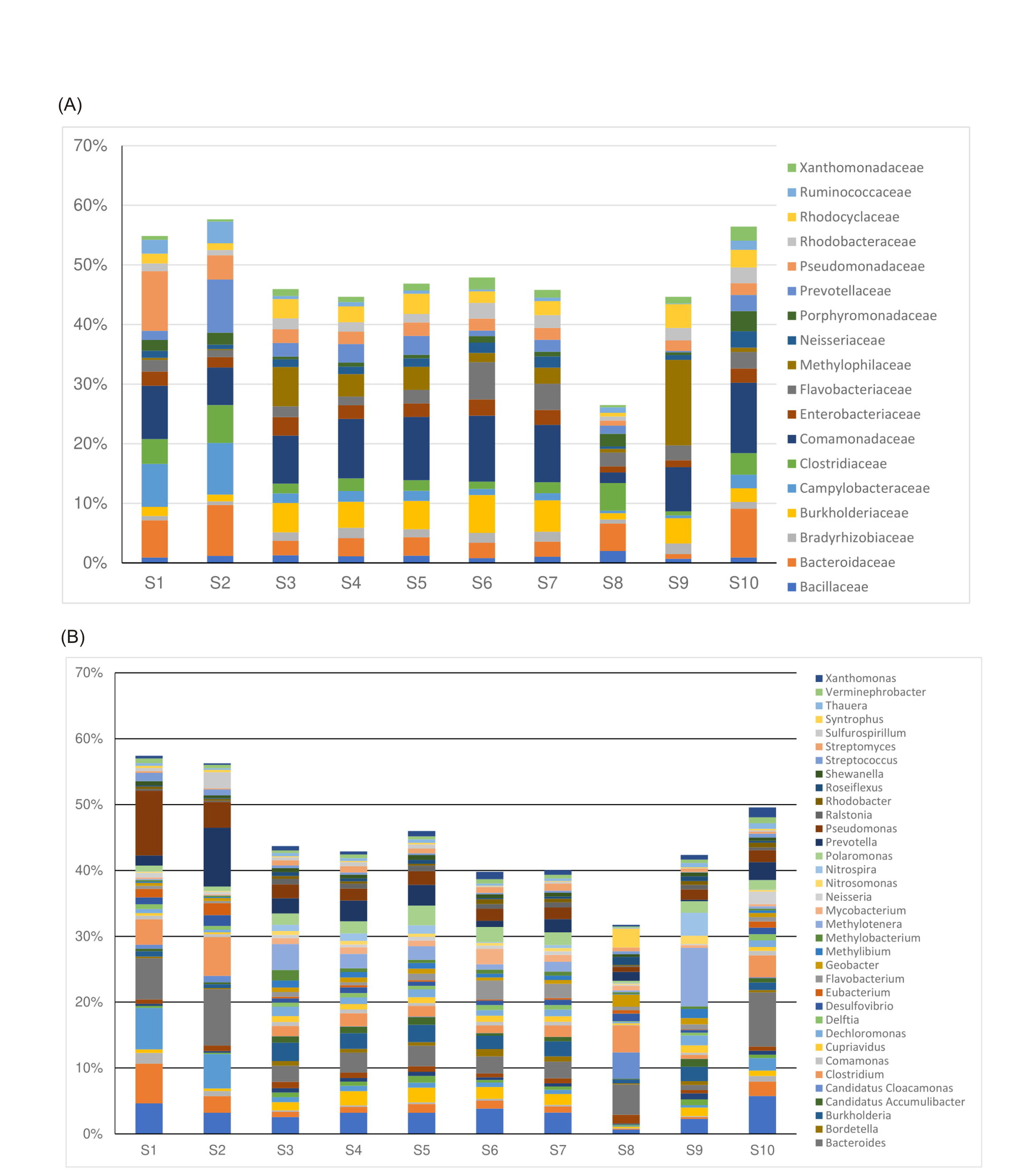

Supplement: S4 Fig — Relative abundance of dominant families constituting more than 1% of the average value (A) and genera constituting more than 0.5% of average value (B) in eubacterial community from different stages of technological process. (TIF) [file pone.0250514.s004.tif]
